# Effect of time window on MVC reference for quantifying spastic cocontraction in spastic paresis

**DOI:** 10.3389/fneur.2026.1703771

**Published:** 2026-03-04

**Authors:** Maud Pradines, Jean-Michel Gracies, Marina Guihard

**Affiliations:** 1 UR 7377 BIOingénierie Tissus Neuroplasticité (BIOTN), Faculté de Santé, Université Paris-Est Créteil, Créteil, France; 2 Service de Rééducation Neurolocomotrice, Hôpitaux Universitaires Henri Mondor, Créteil, France; 3 Neurorehabilitation Department, Bastia Hospital, University of Corsica, Corte, France

**Keywords:** chronic hemiparesis, electromyography, maximal voluntary contraction, spastic cocontraction, window width

## Abstract

**Introduction:**

In hemiparesis, spastic cocontraction is typically quantified by normalizing electromyographic activity to the root mean square (RMS) values obtained during maximal voluntary contraction (MVC) of the cocontracting muscle when it acts as an agonist. However, the selection of the RMS time window and the use of filtering procedures vary widely across studies, limiting comparability. In this study, we evaluated the effects of window size and low-pass filter cutoff frequency (ƒc) on the RMS values obtained during MVC in chronic hemiparesis.

**Methods:**

Participants with stroke-induced hemiparesis and gastrocnemius spasticity (X_V1–GAS_–X_V3–GAS_ >5°) were tested in a seated position on an isokinetic ergometer, with the knee extended and the ankle positioned at 90°. Surface electromyography (EMG) was recorded from the medial and lateral gastrocnemius (MG and LG), soleus (SL), and tibialis anterior (TA) during standardized isometric plantar flexor and dorsiflexor MVCs. RMS values were computed using sliding windows ranging from 5 to 1,250 ms (in increments of 5 ms) and analyzed across low-pass filter cutoff frequencies between 6 and 100 Hz.

**Results:**

A total of 20 participants with hemiparesis (age: 56.4 ± 7.0 years and time since lesion: 7.8 ± 5.7 years) were included. Regardless of muscle type, experimental RMS curves as a function of window size adhered to a first-order model, with high consistency across trials (*R*^2^ ≈ 0.90, RMSE ≈ 8%). RMS values stabilized beyond 168.3 ms (time constant *τ* = 33.6 ms; 5*τ* threshold). In contrast, low-pass filtering caused a marked loss of amplitude, with >40% reduction in RMS magnitude at cutoff frequencies below 10 Hz, despite preservation of signal shape.

**Conclusion:**

These findings demonstrate that reliable EMG normalization in spastic hemiparesis requires a minimal RMS window of approximately 200 ms during MVC and that unsmoothed EMG should be used to preserve signal amplitude. Methodological standardization using these parameters can improve the validity, reproducibility, and comparability of cocontraction indices across studies and may facilitate their application in clinical assessments and rehabilitation research in spastic paresis.

## Introduction

In chronic hemiparesis, active motion is the parameter most closely correlated with function (1). While the loss of maximal active range of motion may be due to agonist paresis (2, 3), there is also massive evidence of significant hindrance through antagonist cocontraction (4–26). Unlike spasticity, which can be clinically quantified through measures such as the angle of catch and the spasticity grade (Tardieu Scale) (27, 28), spastic cocontraction cannot be distinguished from agonist paresis through clinical examination alone and requires experimental quantification.

Normalization methods for measuring coefficients of antagonist activation, or coefficients of cocontraction, vary, making it difficult to compare data (29). It has been shown that the choice of reference value for establishing the cocontraction ratio is critical (29, 30). Among the denominators used in normalization methods, the maximal voluntary contraction (MVC) of the muscle acting as an agonist has been frequently used, both in healthy participants (31–35) and in neurologically impaired populations (18, 22, 23, 25, 36–38). This normalization has been shown to be reliable and does not appear to be affected by contraction mode or joint kinematics (39–41). However, in various investigations aiming to quantify spastic cocontraction using MVC as a reference, the choice of the root mean square (RMS) time window around the peak varied widely—from 20 ms to 3 s (29, 41). In fact, it has been repeatedly shown in healthy participants that the magnitude of processed EMG from isometric MVC decreases as the width of the window around the peak used to calculate the RMS increases (42, 43). We may hypothesize that RMS behavior follows that of a first-order system, in which the output initially rises rapidly before gradually asymptoting toward its maximum, with *5τ* conventionally used as a standard criterion for measurement stability (44). It is unknown to what extent this behavior applies in individuals with motor command impairments, such as chronic hemiparesis. In this population, determining the window threshold beyond which RMS behaves as a reliable measure is particularly important.

In addition, a linear envelope is often generated after bandpass filtering to facilitate the identification of various phases of coordinated movements such as gait (45, 46). In studies evaluating spastic cocontraction in chronic hemiparesis, low-pass cut-off frequencies used to generate linear envelopes have varied between 3 and 30 Hz (29, 45).

In this study, we analyzed the impact of window width on the magnitude of RMS EMG activity from plantar flexor and dorsiflexor MVCs in individuals with post-stroke chronic hemiparesis by comparing experimental data with a theoretical first-order model. We aimed to determine the threshold value sufficient to reach signal stability for this measurement once the model was validated. We also explored the impact of smoothing on that threshold value.

## Methods

### Population

This ancillary study of the randomized controlled clinical trial *EXC-AVC* (ClinTrials n° NCT06790446) was carried out in the Neurorehabilitation Department of Henri Mondor University Hospitals in Créteil, France, with approval from the Ethics Committee *CPP Sud-Est I, 2020-061*. We enrolled a convenience sample of adult participants with chronic hemiparesis, screened from clinic visits in the department and from neighboring private physical therapy offices. All participants provided written consent.

The inclusion criteria were as follows: Hemiparesis due to a single stroke during adulthood (age 18–80 years) occurring more than 6 months before enrolment, gastrocnemius hypo-extensibility (X_V1-GAS_ < 110°; X_V1_, maximal clinical extensibility of the gastrocnemius muscles) (47) and spasticity (Spasticity Angle X_GAS_ = X_V1_-X_V3_ ≥ 5°, Tardieu) (47), and the ability to walk independently over 10 m (Functional Ambulation Category score ≥4). The exclusion criteria were botulinum toxin injections in the triceps surae in the past 3 months before inclusion (48) and concurrent orthopedic disorders affecting the ankle.

### Experimental procedures

**Figure 1:**
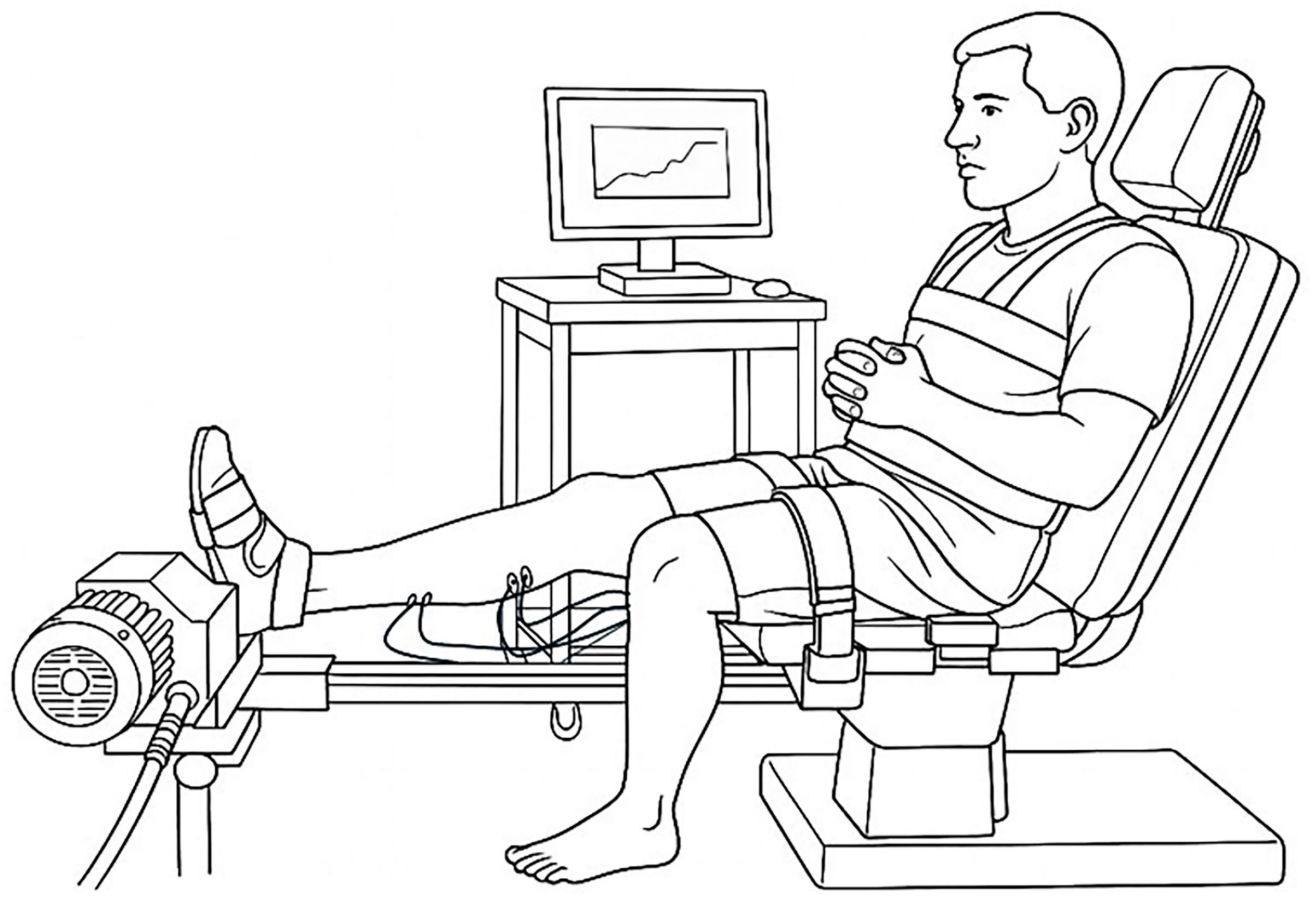
Participant position on the ergometer.

With the participant seated, the skin was abraded and four pairs of surface electrodes were positioned over the soleus (SOL), medial and lateral gastrocnemius (MG and LG, respectively), and tibialis anterior (TA) according to the SENIAM recommendations (49). Participants were then positioned on an isokinetic ergometer (Con-Trex™, Duebendorf, Switzerland) with 60° hip flexion, the knee extended, and the foot strapped to a footplate at 90° X_V1-GAS_ (Figure 1) (18). Maximal voluntary isometric contractions in plantar flexion and dorsiflexion were performed in a standardized manner, including a warm-up effort before the first trial, followed by three successive maximal efforts, each held for approximately 5 s, with two-min rests in between. For each MVC trial, the torque trace was visually inspected, and only segments showing stable torque (±5% variation over ≥500 ms) were retained for RMS computation. Transient fluctuations, the initial ramp-up, and post-fatigue drifts were excluded. RMS sliding windows were applied strictly within these torque-stable segments.

### Data processing

**Figure 2:**
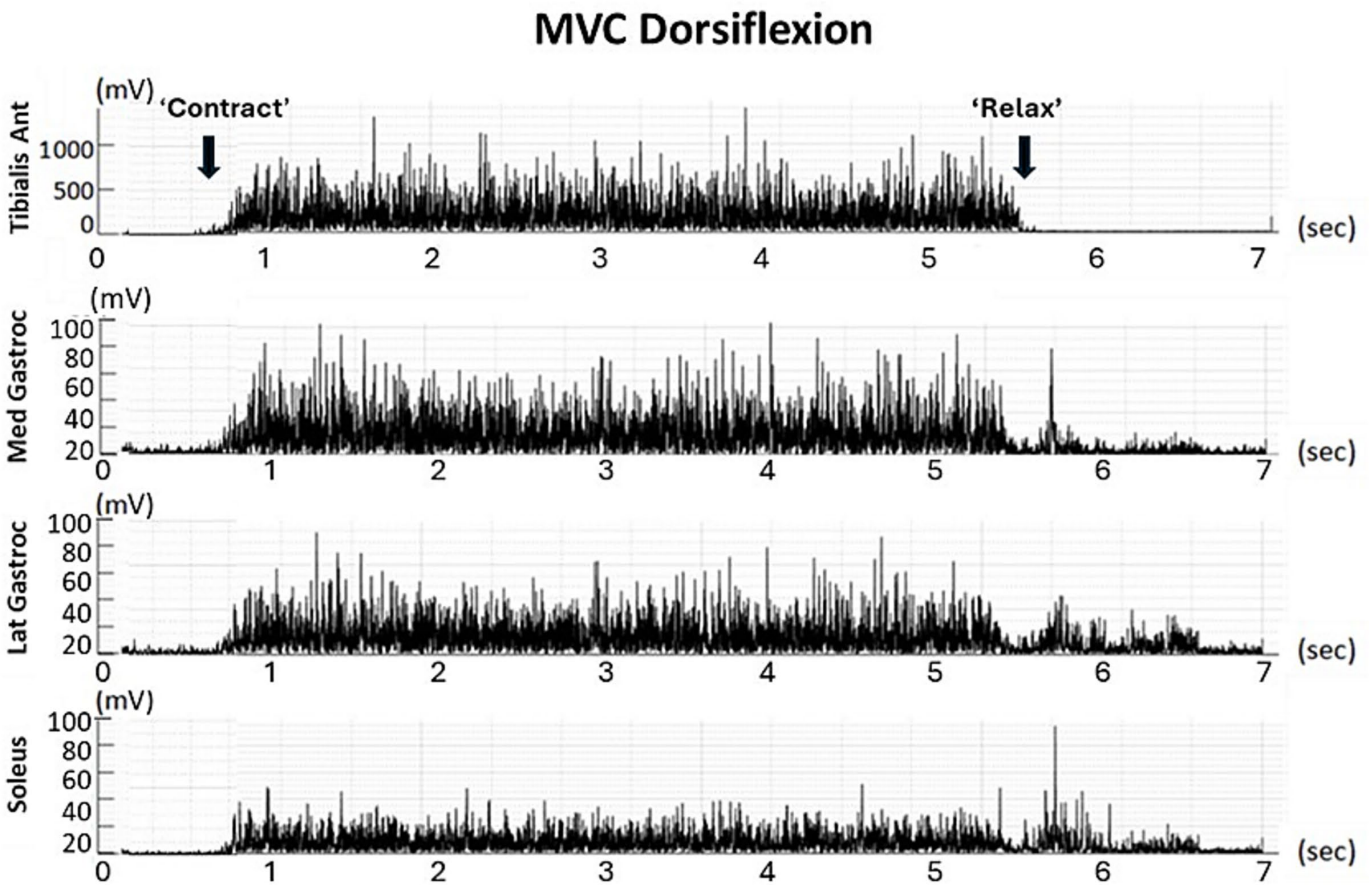
RMS of filtered signals from the four muscles during a single MVC trial in dorsiflexion (individual data).

Electromyographic signals from the SOL, MG, LG, and TA muscles were collected using a ME6000-T16 EMG system (MegaElectronics Ltd., Kuopio, Finland) at a sampling rate of 1,000 Hz, amplified (gain × 1,000), saved to a hard drive, and analyzed offline using MATLAB© (MathWorks, 2023). Signal processing included the following steps: (i) filtering using a fourth-order Butterworth analog band-pass filter (20–450 Hz), (ii) baseline correction by removing the DC offset, and (iii) full-wave rectification (Figure 2). All EMG recordings were visually inspected to identify movement artifacts, electrode–skin contact loss, electrical noise, or saturation. Trials exhibiting artifacts over more than 10% of the torque-stable plateau were discarded and reacquired (<5% of trials). No amplitude normalization was applied between participants beyond RMS windowing during MVC.

A MATLAB script was used to quantify RMS values measured with sliding windows, varying in 5 ms steps starting from 5 ms up to a maximal window width of 1,250 ms. This process yielded RMS values for 250 distinct time windows for each MVC trial of the four targeted muscles in each participant.

Using a second script, independent from the first one, a linear envelope with low-pass filter cut-off frequencies was applied, as MVC can be used to normalize spastic cocontraction during functional tasks such as gait. This script allowed us to determine the changes in the RMS of the targeted muscle activity over time as a function of the smoothing cut-off frequency of the linear envelope. The selection of cut-off frequencies for this analysis was based on a previous reference study, which used seven distinct frequencies: 100, 50, 30, 20, 10, 8, and 6 Hz (45).

### Statistical analysis

Participant characteristics were summarized using descriptive statistics for continuous quantitative variables. To assess how well the model of a first-order system fit the data, goodness-of-fit criteria such as the coefficient of determination (*R*^2^) and the root mean square error (RMSE) were used. *R*^2^ indicated the proportion of variance explained by the model, and the RMSE measured the average difference between the predicted and observed values.

If concordance with the model was confirmed, the time constant (*τ*) was estimated to determine the threshold value beyond which RMS stability was ensured (5*τ*).

Then, the percentage of signal loss (RMS amplitude) was analyzed as a function of the low-pass filter cut-off frequency used for smoothing. The influence of the cut-off frequency on the RMS stability threshold (5*τ*) was investigated.

## Results

### Data characteristics

A total of 20 individuals with chronic hemiparesis were enrolled. The mean age was 56.4 ± 7.0 years, and the time since lesion was 7.8 ± 5.7 years. The three MVC trials for each of dorsiflexion and plantar flexion represented a total of 480 acquisitions from the four muscles: MG, LG, SOL, and TA.

### Comparison of experimental RMS data variation with window width and a first-order theoretical model

**Figure 3:**
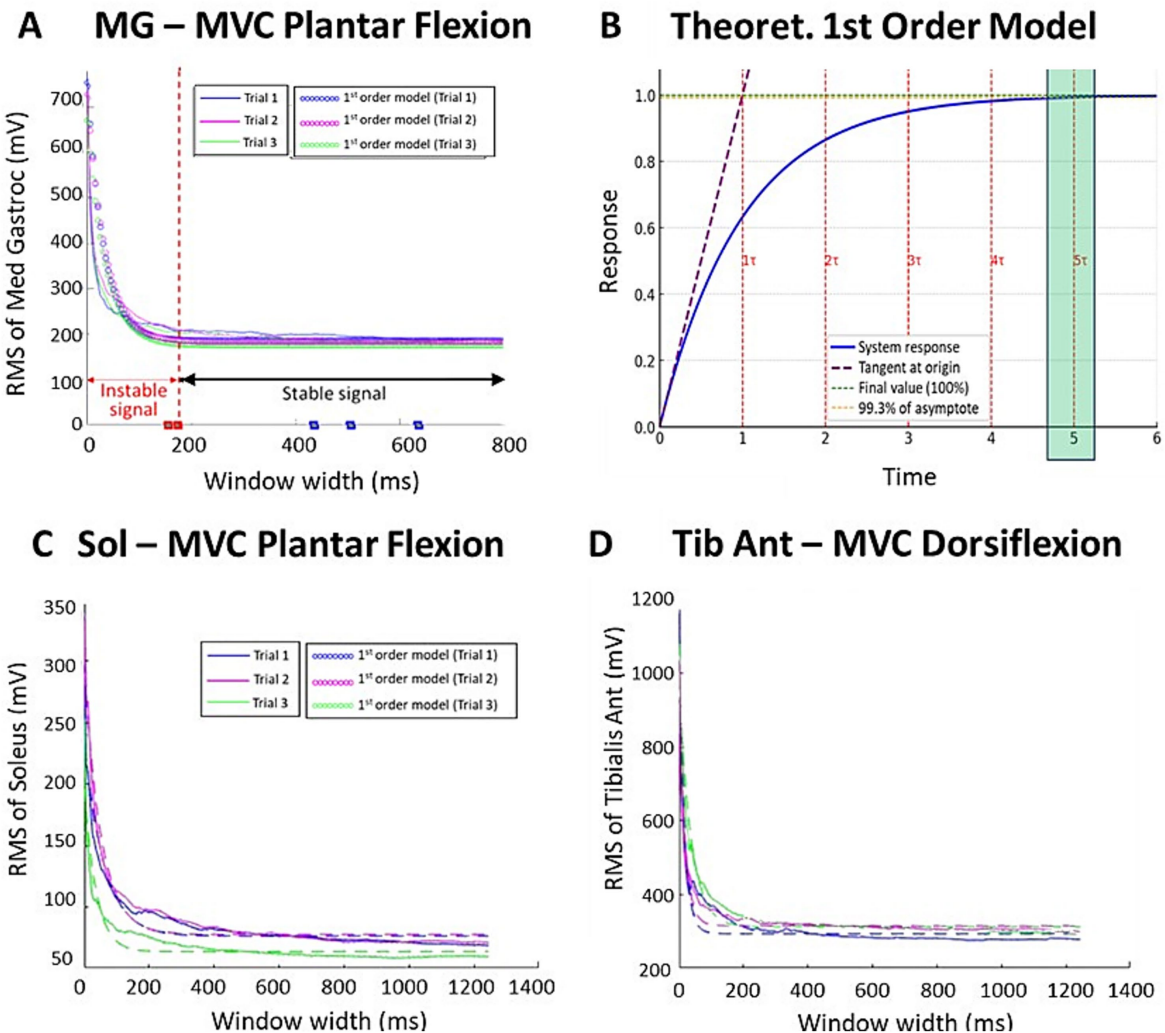
Effect of window width on RMS values during each MVC trial for a single participant: MG **(A)**, SOL **(C)**, and tibialis anterior **(D)**, compared to a first-order model **(B)**.

The window width analysis showed that RMS values followed a first-order system model, with the temporal response described by the following equation: $y(t)=1-e^{-\frac{t}{\tau }}$ (*τ*, time constant; Figure 3). Coefficients of determination *R*^2^ were consistent throughout MVC trials for the targeted muscles, with approximately 90% of the RMS amplitude variation explained by the model (Table 1). Since RMS evolution followed a first-order response, the time constant *τ* could be estimated for each trial, allowing parametric identification of the minimal window width ensuring stable RMS values. In such systems, 5*τ* corresponds to 99.3% of the asymptotic value, providing an objective signal-processing stability threshold. The root mean square error (RMSE) was also consistent across trials and muscles, with an average difference of 8% between predicted and observed values (Table 1).

**Table 1:** Comparisons of theoretical first-order system model trials with experimental data using the coefficients of determination (*R*^2^) and the Normalized Root Mean Square Error (NRMSE) (% difference between predicted and observed values).

Muscle	Concordance determinants	1st trial	2nd trial	3rd trial	Mean
MVC plantar flexion (*N* = 20)					
Gastroc Med	*R* ^2^	0.90 ± 0.03	0.90 ± 0.04	0.90 ± 0.03	0.90 ± 0.03
	NRMSE (%)	8.13 ± 2.62	7.62 ± 1.49	7.65 ± 1.27	7.80 ± 1.79
Gastroc Lat	*R* ^2^	0.90 ± 0.03	0.91 ± 0.03	0.89 ± 0.04	0.90 ± 0.03
	NRMSE (%)	8.44 ± 2.01	8.19 ± 2.04	8.09 ± 1.75	8.24 ± 1.93
Soleus	*R* ^2^	0.90 ± 0.03	0.90 ± 0.04	0.90 ± 0.04	0.90 ± 0.04
	NRMSE (%)	8.49 ± 1.34	8.55 ± 2.42	8.65 ± 2.34	8.57 ± 2.03
MVC dorsiflexion (*N* = 20)					
Tibialis Ant	*R* ^2^	0.90 ± 0.04	0.90 ± 0.05	0.88 ± 0.04	0.90 ± 0.04
	NRMSE (%)	9.33 ± 5.35	7.95 ± 2.53	8.43 ± 2.27	8.57 ± 3.38

### Calculation of the time constant *τ*

The mean time constant *τ* for each MVC trial and for each of the ankle muscles is displayed in Table 2. The time constant τ did not differ significantly across muscles (NS, T-*test*; Table 2). The mean time constant τ across the four muscles and all trials was *τ* = 33.6 ± 18.1 ms. The 5*τ* threshold, above which the signal is considered to have reached a reliable value (estimated at 99.3% of its final value according to the model), was 168.3 ms.

**Table 2:** Time constant (*τ*) characterization and asymptote point (5*τ*).

Time constant *τ*	1st trial		2nd trial		3rd trial		Mean	
	*τ*	5*τ*	*τ*	5*τ*	*τ*	5*τ*	*τ*	5*τ*
MVC plantar flexion (*N* = 20)								
Gastroc Med	35.4 ± 16.9	176.9	30.2 ± 10.1	151.1	28.2 ± 6.1	140.8	31.3 ± 11.0	156.3
Gastroc Lat	31.8 ± 10.5	158.9	40.6 ± 31.6	202.7	36.9 ± 20.6	184.7	36.4 ± 20.9	182.1
Soleus	32.3 ± 13.4	161.3	31.5 ± 15.3	157.7	37.5 ± 17.0	187.3	33.8 ± 15.2	168.8
MVC dorsiflexion (*N* = 20)								
Tibialis Anterior	31.3 ± 11.6	156.8	27.8 ± 10.9	138.9	40.3 ± 52.8	201.8	33.2 ± 25.1	165.8

### Impact of the linear envelope on the threshold window width for reliability

**Figure 4:**
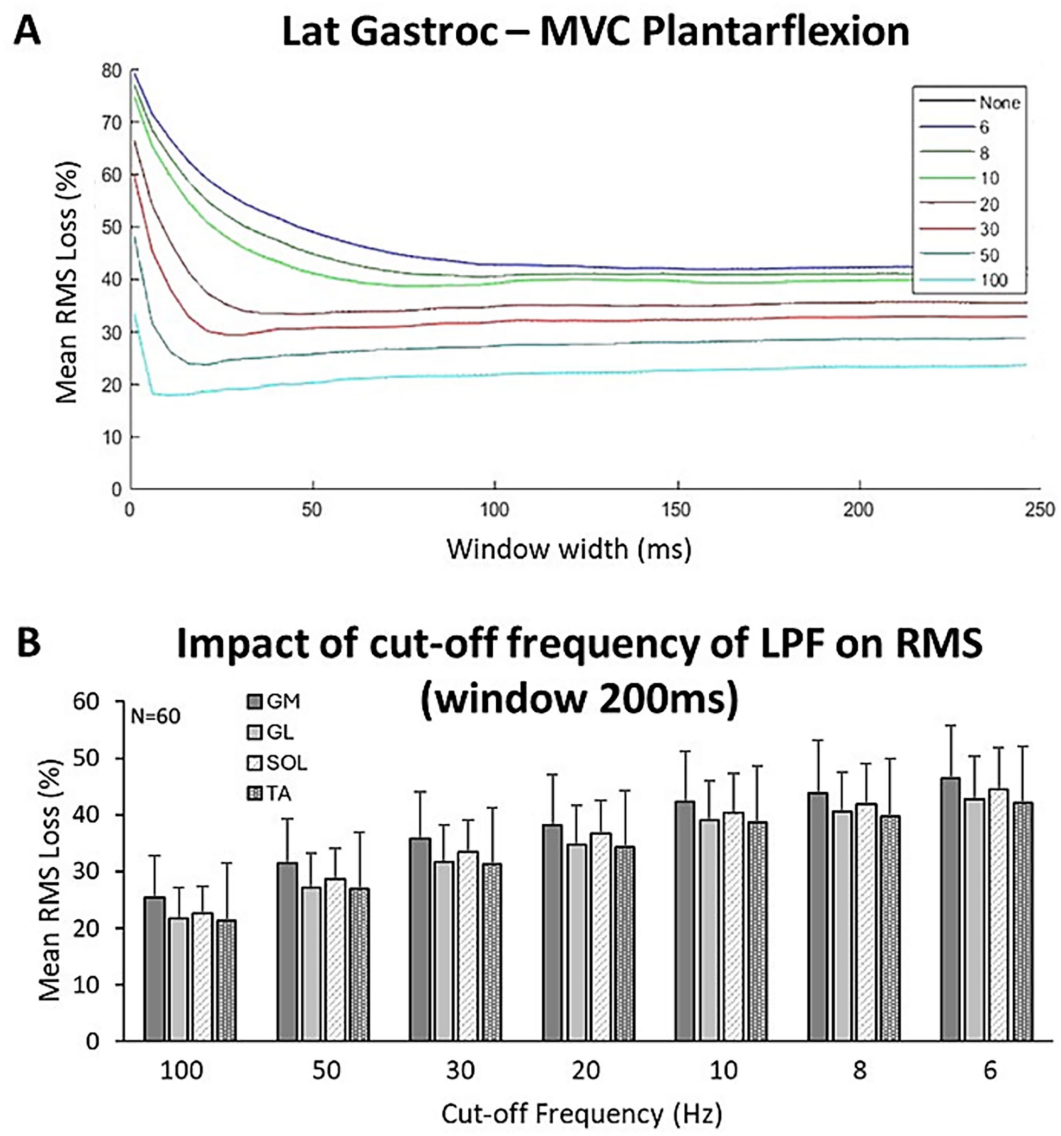
Effect of the filtering process on RMS according to window width for each of the selected cut-off frequencies (Ƒc) **(A)**; impact of Ƒc on RMS loss in each of the four muscles **(B)**.

Percentages of signal loss as a function of the applied cut-off frequency for offline smoothing are displayed in Figure 4. Losses of RMS magnitude (mean of the four muscles) after the smoothing process were 23 ± 6% for a cut-off frequency (Ƒc) of 100 Hz, 28 ± 6% for Ƒc = 50 Hz, 33 ± 7% for Ƒc = 30 Hz, 36 ± 7% for Ƒc = 20 Hz, 40 ± 8% for Ƒc = 10 Hz, 42 ± 8% for Ƒc = 8 Hz, and 44 ± 8% for Ƒc = 6 Hz.

## Discussion

This study was designed as a methodological investigation aimed at determining the minimal RMS window width required to obtain a stable MVC reference for EMG normalization in hemiparetic patients. Our objective was not to analyze cocontraction during gait or dynamic tasks but rather to ensure that the MVC reference used in cocontraction indices is robust and reliably estimated. We demonstrated that activity measurements of isometric plantar flexor and dorsiflexor MVCs in participants with chronic hemiparesis remain stable, provided that window widths used for the measurements are ≥168.3 ms, regardless of the cut-off frequency applied for smoothing. In addition, calculating the RMS on an *unsmoothed* signal helps preserve EMG signal amplitude in this population. These findings should help standardize RMS measurements when normalizing cocontraction evaluations in spastic paresis.

### Should maximal voluntary contraction be used to normalize spastic cocontraction?

Various methods have been employed to normalize raw root mean square (RMS) values for the quantification of spastic cocontraction (26, 28, 39, 40, 49–53). Normalization remains a critical step, particularly because the biological meaning of myoelectrical signals may vary according to electrode locations and joint positions (among many other factors) (53–61). The normalization process allows the comparison of spastic cocontraction quantifications across muscles, individuals, joint positions, and studies, both in healthy participants and in participants with spastic paresis (41, 53–67).

The advantages and limitations of various normalization methods have been widely discussed over the past three decades (41, 58–67). Among these, an alternative to MVC, particularly when maximal contractions cannot be reliably obtained (e.g., due to limited participant cooperation), involves using the maximal compound muscle action potential evoked by peripheral stimulation of the afferent nerve (Mmax) (68–70). However, aside from the discomfort this measurement may cause in individuals with hemiparesis, Mmax may activate muscles beyond the cocontracting muscle, and EMG normalized to Mmax has been shown to be approximately 11 times smaller than EMG normalized to maximal voluntary contraction (68, 70). Therefore, the cocontraction ratio normalized using MVC may more accurately reflect antagonist muscle activation and probably carries greater physiological significance (70). The use of EMG data from maximal voluntary contraction (MVC) has been recommended and endorsed by reference guidelines (71). These guidelines emphasize that EMG normalization for one contractile condition can be effectively achieved using EMG data from another condition. Among the eight EMG standardization methods identified across 26 studies involving healthy participants, Burden also advocated using isometric MVC as a reference (41) A similar recommendation has been made for normalizing cocontraction measurements in individuals with post-stroke hemiparesis, based on considerations of practicality and feasibility (51).

### Identification of the RMS stability threshold for cocontraction analysis in hemiparesis

To the best of our knowledge, this is the first study to formally evaluate the impact of window widths around the peak on measured root mean square (RMS) values during maximal voluntary contraction in individuals with spastic paresis (18, 22, 23, 25, 31–37). Interestingly, in healthy participants, McLean et al. demonstrated that window widths exceeding 200 ms of EMG activity during maximal voluntary contraction yielded stable estimates (42), facilitating comparisons across studies that employed various window widths for RMS computation. Until now, there has been no consensus regarding the most appropriate RMS windowing approach in spastic paresis (29, 30). Prior research has utilized time windows of approximately 500 ms around the peak, both in healthy individuals (35, 72) and in individuals with hemiparesis (18, 25), while 100-ms windows have also been employed (11, 20, 23). This variability in window durations may have compromised the comparability of the cocontraction ratios obtained. Although cocontraction indices were not directly computed in this methodological study, we assume that instability in the denominator can substantially affect the resulting value. The influence of window width on the ratio becomes particularly evident for windows shorter than the stability threshold. To illustrate this point qualitatively, we performed a simple simulation based on the example shown in Figure 3. When the antagonist RMS during cocontraction was kept constant at its 200-ms value, varying only the RMS MVC window in the denominator yielded cocontraction estimates of 0.12, 0.09, 0.075, 0.06, and 0.045 for window widths of 200 ms, 100 ms, 50 ms, 25 ms, and 10 ms, respectively. This demonstrates that using an unstable MVC window can reduce the estimated cocontraction value by more than half compared to a stable 200-ms reference. Conversely, when the same window width was applied to both the numerator and denominator, the ratios remained much closer (between 0.11 and 0.07), suggesting that consistent windowing can mitigate, but not fully eliminate, the impact of window duration on ratio stability. Accordingly, RMS windows of ≥200 ms during MVC provide a sufficiently stable reference value, thereby limiting denominator-driven fluctuations in cocontraction ratios and improving the reliability of ratio-based cocontraction metrics.

### Toward standardization of an index to quantify cocontraction in spastic paresis

In individuals with stroke or cerebral palsy, methods employed to measure spastic cocontraction during gait have been characterized by considerable variability, preventing authors from recommending the most appropriate methodology (28, 62, 73). This variability extends beyond data acquisition; EMG signals have been normalized using diverse temporal and amplitude parameters. Temporal normalization methods have used periods such as 5% of the gait cycle duration (35, 61), 100% of the step cycle duration (73), the mean cycle duration, and 100% of the gait cycle duration (37, 74–78). Amplitude normalization methods have included the mean amplitude (17) or peak value of each gait cycle (35, 36, 73), the mean amplitude of three gait cycles (79), and mean or maximal voluntary contraction (MVC) values (35, 36, 73).

In addition, various strategies to calculate cocontraction indices have been utilized (29). Falconer and Winter proposed quantifying spastic cocontraction using a reference value that combined agonist and antagonist muscle activation capacities (50). However, such a mathematical construction ignores the lack of comparability between EMG signals coming from two different muscles, that is, the different biological meanings of myoelectric signals detected on one side of the joint and those detected on the other (80). During the stance phase, post-stroke participants did not exhibit significant excessive antagonist cocontraction based solely on this type of antagonist–agonist cocontraction index (73). Conversely, studies that defined the cocontraction ratio as antagonist RMS activity divided by the RMS activity of *the same muscle acting as an agonist* (an index of *antagonist activation*) have clearly observed excessive cocontraction, particularly when the cocontracting muscle is stretched (10, 18, 23, 25, 81).

Finally, a variety of procedures have been employed to generate a smoothed linear envelope (LE) and to minimize variability, thereby enhancing the repeatability of electromyography profiles (81–83). A broad spectrum of smoothing parameters, specifically low-pass filters, have been utilized in studies involving hemiparetic individuals during gait analysis: 3 Hz (36, 84), 6 Hz (85), 10 Hz (17), 20 Hz (37, 76, 86), and 25 Hz (77, 78, 88). However, it has been suggested that a cut-off frequency below 8.9 Hz should be avoided when investigating cocontraction during gait, as this may result in the loss of relevant information in the envelope signal (44), a finding confirmed by the present study. Indeed, signal loss exceeded 40% at a cut-off frequency below 10 Hz. As the cocontraction index is defined as a ratio, low-pass filtering would be expected to affect antagonist and agonist RMS amplitudes in a broadly proportional manner, thereby exerting only a limited effect on the ratio itself. However, by compressing the overall amplitude range, heavy smoothing reduces measurement resolution and may, nonetheless, alter the cocontraction estimate. Therefore, once the phases of a movement have been precisely identified using a low-pass filter, we suggest reverting to an unsmoothed signal to calculate the RMS to preserve signal quality and quantity, particularly in individuals with hemiparesis, whose EMG signals are altered compared to healthy individuals (87).

### Limitations

This study is limited by its focus on isometric plantar flexor and dorsiflexor MVCs and by its single-center design. As the objective was solely to determine the window width required to obtain a stable MVC-based reference value for ratio normalization in an isometric context, the recommendations presented here apply only to this methodological scope. Although the present study standardizes the reference value that may later be used to normalize cocontraction ratios in gait or dynamic tasks, the stability of RMS estimates for the numerator under dynamic conditions remains to be investigated. Finally, cross-talk can never be completely excluded when using surface EMG (59); however, several elements suggest that it is unlikely to be the primary source of the antagonist activity recorded in our participants. First, electrode placement strictly followed the SENIAM recommendations, and the gastrocnemius medialis, gastrocnemius lateralis, and soleus muscles are anatomically distant from the tibialis anterior, which substantially reduces the likelihood of cross-talk contamination. Second, the antagonist EMG exhibited muscle-specific amplitude and temporal patterns rather than the uniform attenuation expected from tibialis anterior signal leakage. Third, the presence of antagonist coactivation has been consistently reported in hemiparetic patients (10, 18, 23, 25, 81), supporting the interpretation that the antagonist EMG observed here reflects genuine cocontraction rather than artefactual spillover. Nonetheless, as with any surface EMG investigation, we acknowledge that a residual contribution of cross-talk cannot be entirely ruled out.

## Conclusion

To normalize the measurement of spastic cocontraction in spastic paresis, an optimal time window of 200 ms during maximal voluntary contractions may be recommended to determine the reference RMS. Although using a low-pass filter can help qualitatively determine the phases of a movement, it proves highly deleterious for signal quantification, with a significant loss of 40% of the RMS signal at cut-off frequencies below 10 Hz, when seeking to quantify cocontraction indices, which may be best measured using unfiltered EMG signals.

## Data Availability

The raw data supporting the conclusions of this article will be made available by the authors without undue reservation.
